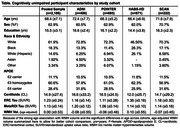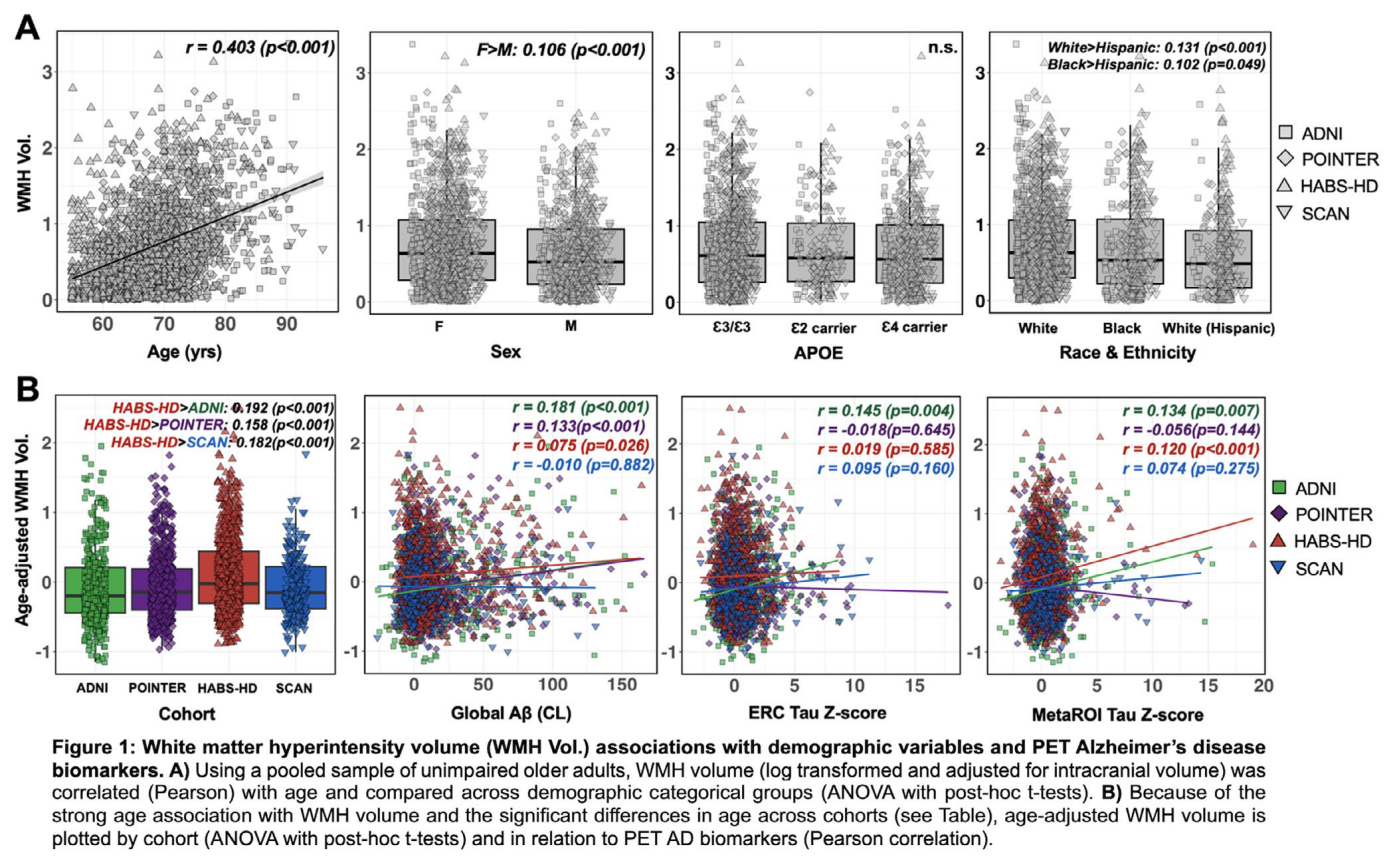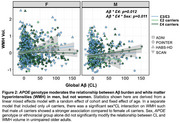# White matter hyperintensities and Alzheimer's disease pathology in heterogeneous cohorts

**DOI:** 10.1002/alz70856_100135

**Published:** 2025-12-24

**Authors:** Theresa M. Harrison, Yishu Chao, Trevor A Chadwick, Jacinda Taggett, Pauline Maillard, Charles Decarli, Susan M. Landau, William J. Jagust, for the U.S. POINTER Study Group, for the Alzheimer's Disease Neuroimaging Initiative

**Affiliations:** ^1^ University of California, Berkeley, Berkeley, CA, USA; ^2^ University of California, Davis, Davis, CA, USA; ^3^ Neuroscience Department, University of California, Berkeley, Berkeley, CA, USA; ^4^ Lawrence Berkeley National Laboratory, Berkeley, CA, USA

## Abstract

**Background:**

White matter hyperintensities (WMH) are considered a biomarker of cardiovascular disease, but may also be associated with Alzheimer's disease (AD) pathology. We performed this study to investigate how WMH are related to AD biomarkers and key demographic and genetic factors in a number of heterogeneous cohorts.

**Method:**

PET and MR scans from cognitively normal older adults aged >55 in ADNI, the Health and Aging Brain Study‐Health Disparities (HABS‐HD), POINTER Imaging and Standardized Centralized Alzheimer's & Related Dementias Neuroimaging (SCAN) studies were processed using harmonized pipelines. Total WMH volume was calculated in native space with 3D T1 and FLAIR MRI, log‐transformed and corrected for head size. Global beta‐amyloid (Aβ) burden was quantified in centiloids (CLs). Tau‐PET SUVRs were z‐scored using tracer‐specific reference cohorts of cognitively normal, Aβ‐ adults aged 60‐70yrs. Key demographic and genetic data (age, sex, *APOE* genotype and ethnoracial group) were obtained for each cohort. Associations between WMH volume and demographic variables, and moderators of the relationship between WMH volume and Aβ were explored in the pooled sample and within cohort.

**Result:**

2,186 unimpaired older adults were included across 4 cohorts which differed on several characteristics (Table). Greater WMH volume was associated with older age, female sex and ethnoracial group (non‐Hispanic White>Black>Hispanic White) but not *APOE* genotype (Figure 1A). Compared to other cohorts, HABS‐HD and POINTER Imaging had the highest age‐adjusted WMH volumes and all cohorts except SCAN showed significant associations between age‐adjusted WMH volume and CLs (Figure 1B). Associations between WMH volume and measures of tau were less consistent across cohorts. In the pooled sample, a three‐way interaction revealed that *APOE* genotype moderates the relationship between CLs and WMH volume (e4>e3>e2) in men, but not women (Figure 2). There were no other significant moderation effects of sex, *APOE* genotype or ethnoracial group on the relationship between CLs and WMH.

**Conclusion:**

Total WMH volume is related to demographic characteristics and Aβ burden, but it remains unclear how AD‐related pathology interacts with participant characteristics to drive white matter disease. Next steps include investigating regional WMH volume, especially posterior regions which we predict will show stronger associations with Aβ burden.